# Involvement of Calcium-Dependent Pathway and β Subunit-Interaction in Neuronal Migration and Callosal Projection Deficits Caused by the Cav1.2 I1166T Mutation in Developing Mouse Neocortex

**DOI:** 10.3389/fnins.2021.747951

**Published:** 2021-12-08

**Authors:** Nao Nakagawa-Tamagawa, Emi Kirino, Kohtaroh Sugao, Hidetaka Nagata, Yoshiaki Tagawa

**Affiliations:** ^1^Department of Physiology, Graduate School of Medical and Dental Sciences, Kagoshima University, Kagoshima, Japan; ^2^Laboratory for Molecular Analysis of Higher Brain Function, RIKEN Center for Brain Science, Saitama, Japan; ^3^Platform Technology Research Unit, Sumitomo Dainippon Pharma Co., Ltd., Osaka, Japan

**Keywords:** Timothy syndrome, calcium channel, radial migration, callosal projection, neocortex

## Abstract

**Introduction:** Gain-of-function mutations in the L-type Ca^2+^ channel Cav1.2 cause Timothy syndrome (TS), a multisystem disorder associated with neurologic symptoms, including autism spectrum disorder (ASD), seizures, and intellectual disability. Cav1.2 plays key roles in neural development, and its mutation can affect brain development and connectivity through Ca^2+^-dependent and -independent mechanisms. Recently, a gain-of-function mutation, I1166T, in Cav1.2 was identified in patients with TS-like disorder. Its channel properties have been analyzed *in vitro* but *in vivo* effects of this mutation on brain development remain unexplored.

**Methods:** *In utero* electroporation was performed on ICR mice at embryonic day 15 to express GFP, wild-type, and mutant Cav1.2 channels into cortical layer 2/3 excitatory neurons in the primary somatosensory area. The brain was fixed at postnatal days 14–16, sliced, and scanned using confocal microscopy. Neuronal migration of electroporated neurons was examined in the cortex of the electroporated hemisphere, and callosal projection was examined in the white matter and contralateral hemisphere.

**Results:** Expression of the I1166T mutant in layer 2/3 neurons caused migration deficits in approximately 20% of electroporated neurons and almost completely diminished axonal arborization in the contralateral hemisphere. Axonal projection in the white matter was not affected. We introduced second mutations onto Cav1.2 I1166T; L745P mutation blocks Ca^2+^ influx through Cav1.2 channels and inhibits the Ca^2+^-dependent pathway, and the W440A mutation blocks the interaction of the Cav1.2 α1 subunit to the β subunit. Both second mutations recovered migration and projection.

**Conclusion:** This study demonstrated that the Cav1.2 I1166T mutation could affect two critical steps during cerebrocortical development, migration and axonal projection, in the mouse brain. This is mediated through Ca^2+^-dependent pathway downstream of Cav1.2 and β subunit-interaction.

## Introduction

Autism spectrum disorder (ASD) is a neurodevelopmental disease. Its etiology has been extensively studied but remains largely unknown, primarily because the developing brain undergoes complex processes influenced by many genetic and environmental factors (Abrahams and Geschwind, 2008; Courchesne et al., 2020). However, there are some cases where a single mutation in a certain gene is linked to ASD. One such case is a gain-of-function mutation, G406R, in the L-type Ca^2+^ channel Cav1.2 that causes Timothy syndrome (TS). TS is a multisystem disorder associated with long QT syndrome (LQTS type 8) in the heart, syndactyly, and neurologic symptoms, including ASD, seizures, and intellectual disability (Splawski et al., 2004). G406R mutation alters ion channel kinetics, leading to an abnormal Ca^2+^ overload and Ca^2+^-dependent and -independent gene expression in neuronal cells, which are thought to be the basis for the disease (Paşca et al., 2011; Marcantoni et al., 2020).

Recently, a mutation, I1166T, in Cav1.2 was identified in patients with TS-like disorder (Boczek et al., 2015; Wemhöner et al., 2015). Among the mutations reported in those studies, the patient with I1166T mutation had brain symptoms. The G406R (the original mutation) and I1166T mutations are considered to be gain-of-function; both elicit excess Ca^2+^ influx. Because G406R was the original mutation identified in TS patients, many *in vitro* and *in vivo* studies have been conducted on the G406R mutant channel. As for the I1166T mutant, channel properties have been thoroughly analyzed *in vitro* (Boczek et al., 2015; Wemhöner et al., 2015), but *in vivo* effects of this mutation on brain development have not been reported.

The Cav1.2 Ca^2+^ channel plays important roles in neural development (Krey et al., 2013; Kabir et al., 2017; Horigane et al., 2019). Ca^2+^ influx through the channel and the subsequent intracellular Ca^2+^ signaling are essential for neurodevelopmental processes, such as neurogenesis, migration, and neurite morphogenesis in the neocortex (Kamijo et al., 2018; Panagiotakos et al., 2019). One of the key molecules downstream of Cav1.2 is calmodulin (CaM). Ca^2+^-CaM binds to the C terminus of the Cav1.2 α_1_ subunit and modulates channel activity. Ca^2+^-CaM also activates kinases (such as CaMK) and phosphatases (such as calcineurin; CaN) in the cytoplasm and eventually regulates Ca^2+^-dependent gene expression in the nucleus (Deisseroth et al., 2003; Yap and Greenberg, 2018). However, recent studies suggested another downstream signaling pathway—the Cav1.2 β subunit-dependent pathway (Servili et al., 2018)—which depends on extracellular Ca^2+^ and Ca^2+^ occupancy of the open channel but is Ca^2+^-influx independent. Thus, there are at least two important signaling pathways downstream of Cav1.2 (Krey et al., 2013; Servili et al., 2018).

In this study, we asked two questions. First, what is the effect of the I1166T mutant channel expression on neocortical development? We expressed the I1166T mutant channel in *in vivo* mouse cortical neurons by *in utero* electroporation, and found that neuronal migration was impaired and callosal axon projections were disturbed. Second, which signaling pathway is involved in the defects of migration and callosal projections caused by the I1166T mutant? We attempted to assess the contribution of Ca^2+^ influx-dependent and β subunit-dependent pathways downstream of the Cav1.2 I1166T mutant channel to the circuit formation deficits. We found that the Ca^2+^ influx-dependent pathway surely contributed to the phenotypes in brain development and that the interaction of the Cav1.2 α_1_ subunit to the β subunit was also involved in the deficits caused by the I1166T mutation. Thus, our findings suggest that the Cav1.2 I1166T mutation affects brain development and connectivity through Ca^2+^-dependent pathway and β subunit-interaction.

## Materials and Methods

### Mice

All experiments were approved by the Animal Experiments Committee and the Genetic Recombinant Experiment Safety Committee of Kagoshima University and performed according to the guidelines of the university. Pregnant ICR mice were purchased from Japan SLC and used in all experiments.

### Plasmid Construction

**FIGURE 1:**
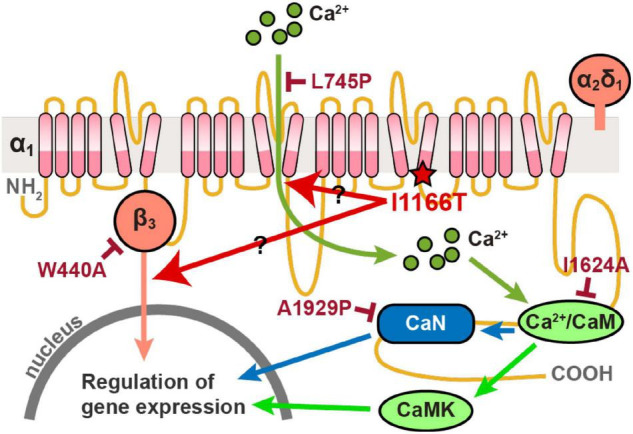
Diagram of signal pathways downstream of Cav1.2 that regulate gene expression. The I1166T and four additional mutations used in this study are also shown.

All plasmid vectors for the expression of the human Cav1.2 α_1_ subunit of the wild-type (WT) and all mutants, α_2_δ_1_ subunit, and β_3_ subunit were synthesized by Genscript. The Cav1.2 channel subunit sequences were under the chicken-actin-globin (CAG) promoter and followed by the WPRE and BGH polyA signal. The enhanced green fluorescent protein (EGFP) was expressed by pCAsalEGFP vector (Bando et al., 2014). The signal pathways downstream of Cav1.2 that regulate gene expression, and the I1166T and four additional mutations used in this study are shown in Figure 1.

### *In utero* Electroporation

*In utero* electroporation was performed as reported previously (Saito and Nakatsuji, 2001; Tabata and Nakajima, 2001). In brief, all the plasmid vectors were prepared at 1 μg/mL with 0.2 μg/mL Fast Green FCF (Nacalai Tesque) in 1/10 Tris-EDTA Buffer. To express the α_1_ subunit of the WT and all mutant Cav1.2 channels, α_2_δ_1_ and β_3_ subunits, and EGFP were expressed together otherwise noted. For the GFP-alone control experiments, EGFP was expressed alone. ICR mice at embryonic day 15 (E15) were anesthetized with an intraperitoneal injection of pentobarbital sodium salt (64.8 mg/mL, Tokyo Chemical Industry) in saline and maintained with isoflurane (0.7–1.2%, Forane). The mouse uteruses were exposed, and approximately 0.5–1 μL of DNA solution was injected into the lateral ventricle. The embryo heads were pinched with a tweezer CUY650P5 (Nepa Gene), and five electric pulses of 50 V for 50 ms at 1 Hz were applied using an electroporator NEPA21 (Nepa Gene) or an electroporator CUY21 (BEX). We introduced plasmid vectors into neurons in the middle part of the anterior-posterior axis in the neocortex, centered by the primary somatosensory area (S1). One section per mouse was used for all the analyses. The number of *in utero* electroporation performed for each test group was shown in Supplementary Figure 1.

### Fixation and Sectioning

The pups at postnatal days 14–16 were perfused transcardially with ice-cold 0.1 M phosphate-buffered saline (PBS), followed by fixation with 4% paraformaldehyde (PFA) in 0.1 M phosphate buffer. After the brains were dissected, they were further fixed with PFA overnight at 4°C, and then incubated in 20% sucrose in 0.1 M PBS overnight at 4°C. Coronal sections of 80 μm were cut using a microtome REM-710 (Yamato Kohki) and were mounted on glass slides and embedded in Vectashield with DAPI (Vector).

### Microscopy

All the fluorescent images were acquired using a confocal laser-scanning microscope TCS SP8 (Leica) with a 10 × dry objective. Z-stack images of the slices were acquired in a 2.5 μm-interval.

### Migration Analysis

GFP-positive cells were imaged in the S1 barrel field of the electroporated hemisphere. The three-dimensional positions of cells were determined manually using a custom-made program in MATLAB (MathWorks). A “radial index,” defined as the radial position of the lower edge of white matter, was set to zero and that of pia was set to 1, and a radial index of each neuron was determined. A histogram of cell density along the radial axis was obtained by dividing the cortex into 20 bins and calculating the fraction of GFP-positive cells in each bin. The cells were also categorized into layer 2/3 (“L2/3”), white matter (“WM”), and in between (“Middle”) as follows: the majority of cells were clustered in the upper layer in all samples and were categorized as L2/3, cells located in the white matter were categorized as WM, and cells located between the upper cluster and the white matter were categorized as Middle. WM had a radial index of 0–0.1. The cell numbers analyzed per animal were; GFP-alone, 220.0 ± 71.2; Cav1.2^WT^, 160.0 ± 55.2; Cav1.2^I1166T^, 232.9 ± 108.7; Cav1.2^I1166T/L745P^, 211.9 ± 118.2; Cav1.2^I1166T/W440A^, 181.0 ± 51.3; Cav1.2^I1166T/L745P/E363A/E1115A^, 161.0 ± 74.6; Cav1.2^I1166T/I1624A^, 128.7 ± 57.2; Cav1.2^I1166T/A1929P^, 162.3 ± 46.8; Cav1.2^WT^ α1-only, 308.1 ± 94.5; Cav1.2^I1166T^ α1-only, 275.9 ± 53.0 (Mean ± SD).

### Callosal Projection Analysis

The GFP signals of callosal axons were imaged at the border between primary and secondary somatosensory area (S1/S2 border), where callosal axons arbor densely, contralateral to the electroporated hemisphere. The intensity distribution of background signal was fitted by a Gaussian function and median + 3 × standard deviation of the Gaussian curve was defined as a threshold, and each pixel was determined to have axonal signal (1) or not (0) by whether it had signal intensity higher than the threshold or not. The binarized images of 500 μm width were averaged tangentially to obtain the proportion of pixels with an axonal signal in each radial position. A strong GFP signal was seen with a radial index of 0.4–0.6 and 0.8–1.0, which may correspond to layer 5 and upper layers where callosal axons are arborized densely (Mizuno et al., 2007); therefore, they were assigned “L5” and “L1–3,” respectively. Because the proportion in the white matter, which represents the amount of GFP-positive axons that reached the S1/S2 border, varied between test groups and between samples, the proportion was normalized by the maximum proportion in the white matter and compared between test groups. Several samples had a proportion of WM that was too small (<0.2) to analyze the change along the radial axis; these were excluded from the analysis.

### White Matter Projection Analysis

Axonal GFP signals in the white matter were imaged from the ipsilateral to the contralateral hemisphere under the same scanning conditions. To analyze the axonal GFP signal in the white matter, images of 200 μm × 50 μm (radial axis × tangential axis) centered with the white matter were taken at the ipsilateral area (“Ipsi”), midline (“Midline”), and two contralateral areas (“Contra” and “Contra2;” Contra2 is twice the distance from Midline than Contra). Ipsi and Contra positions were determined where white matter was horizontal. GFP signals were extracted by subtracting the background signal intensity (median + 2 × standard deviation of the Gaussian curve fitted the intensity distribution of background signal) from the images. GFP intensities in each image were normalized with that in the Ipsi area and compared between test groups. To compare the broadness of axon bundles in the white matter, GFP images were averaged in the tangential axis, and Gaussian fit was conducted, and the standard deviations of Gaussian curves were compared between test groups. The few samples in which the Gaussian fit was not appropriately conducted in either Ipsi, Midline, or Contra were excluded from the analysis of bundle broadness. All the analyses of migration, callosal projection, and white matter projection were conducted using MATLAB.

### Statistical Analysis

Error bars represent the standard error of the mean (SEM). Mann-Whitney-Wilcoxon tests with Holm-Bonferroni correction were used to compare test groups. The statistical analyses were conducted using MATLAB.

## Results

### The I1166T Mutation Elicits a Migration Deficit

**FIGURE 2:**
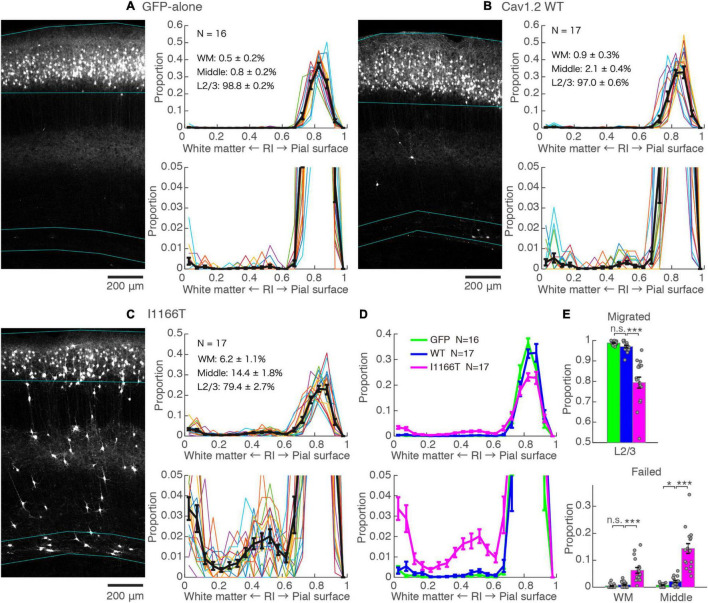
Expression of the Cav1.2 I1166T mutant caused neuronal migration deficit. **(A)** Left, GFP images of electroporated cortex in the GFP-alone group. Cyan lines represent the top; pial surface, the bottom edge of the GFP-positive neuron cluster, and the top and bottom edge of white matter (see section “Materials and Methods”). Right, histogram of radial index (RI) of the GFP-positive neurons. Colors, each sample. Black, mean ± SEM. Y-axis is enlarged and shown at the bottom. **(B,C)** Images and histograms of the Cav1.2^WT^ group **(B)** and Cav1.2^I1166T^ group **(C)**, as shown in **(A)**. **(D)** Histogram of the mean ± SEM are merged for GFP-alone, Cav1.2^WT^, and Cav1.2^I1166T^ groups. **(E)** Proportion of correctly migrated neurons (top) and migration-failed neurons in WM and Middle (bottom). Independent values and mean ± SEM are shown. ***P < 0.001; *P < 0.05; n.s., *P* ≥ 0.05.

An *in utero* electroporation-mediated gene transfer was performed at E15, an age when efficient and specific gene transfer into cortical L2/3 neurons has been reported (Saito and Nakatsuji, 2001; Tabata and Nakajima, 2001). At P14–16, almost all neurons expressing GFP-alone migrated to the upper layer and distributed in a band (98.8 ± 0.2%, *N* = 16, Figure 2A). For neurons with forced expression of Cav1.2^WT^, though a small proportion of neurons failed to migrate (3.0 ± 0.6%, *N* = 17; vs. GFP-alone, *P* = 0.072), neurons migrated primarily to the upper layer (Figures 2B,D,E). On the other hand, a considerable number of neurons with a forced expression of Cav1.2^I1166T^ failed to migrate to the upper layer (20.6 ± 2.7%, *N* = 17) and were scattered across the full depth of the cortex (Figure 2C). These neurons were located relatively densely in the white matter and at the middle of the cortical depth (radial index = 0.5, Figures 2C–E). The ratio of migration-failed neurons in the Cav1.2^I1166T^ group was larger than that of the Cav1.2^WT^ group (*P* = 1.2e−5; Figures 2D,E). These results indicate that the I1166T mutation causes migration failure.

### Calcium-Dependent Pathway and β Subunit-Interaction Are Responsible for the Migration Deficit Caused by Cav1.2^I1166T^ Expression

**FIGURE 3:**
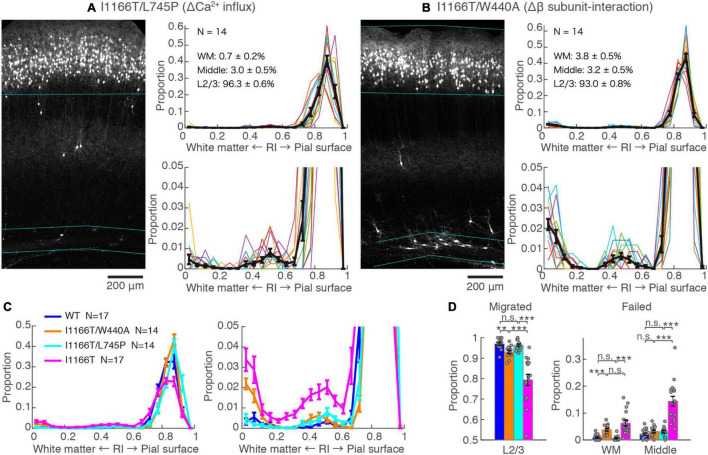
Blockade of Ca^2+^ influx and β subunit-interaction improves migration deficit caused by the I1166T mutation. **(A,B)** GFP images and histograms of the Cav1.2^I1166T/L745P^ **(A)** and Cav1.2^I1166T/W440A^ group **(B)**, as shown in Figure 2A. **(C)** Histogram of the mean ± SEM are merged for Cav1.2^WT^, Cav1.2^I1166T/W440A^, Cav1.2^I1166T/L745P^, and Cav1.2^I1166T^ groups. **(D)** Proportion of correctly migrated neurons (left) and that of migration-failed neurons located in WM and Middle (right). Independent values and mean ± SEM are shown. ***P < 0.001; **P < 0.01; n.s., *P* ≥ 0.05.

The I1166T mutation of Cav1.2 elicits excess Ca^2+^ influx, which has been thought to be the cause of the brain phenotypes in the patients (Boczek et al., 2015; Wemhöner et al., 2015). Therefore, we introduced the second mutation, L745P, that inhibits Ca^2+^ influx (Servili et al., 2018) onto Cav1.2^I1166T^ and examined migration. Neurons expressing Cav1.2^I1166T/L745P^ showed almost normal migration (96.3 ± 0.6%, *N* = 14; vs. I1166T, *P* = 6.0e−5; Figures 3A,C,D), which was equivalent to neurons expressing Cav1.2^WT^ (*P* = 0.55, Figure 3D). This result suggests that Ca^2+^-dependent pathway is responsible for the migration deficit caused by the I1166T mutation. In addition, another TS mutation, G406R, has been reported to enhance gene expression under a calcium-independent, β subunit-dependent pathway (Servili et al., 2020). Therefore, we introduced another mutation, W440A, that inhibits the interaction of Cav1.2 α_1_ subunit to the β subunit. We expected that W440A mutation would inhibit a β subunit-dependent signaling pathway, as suggested in other studies (Berrou et al., 2002; Servili et al., 2018; Yang et al., 2019) (but for a possible effect of this mutation on channel trafficking, please see section “Discussion”). Though the fraction of Cav1.2^I1166T/W440A^-expressing neurons in the upper layer (93.0 ± 0.8%, *N* = 14) was smaller than that of Cav1.2^WT^-expressing neurons (*P* = 0.0041, Figure 3D), migration proceeded normally compared with that of Cav1.2^I1166T^-expressing neurons (*P* = 4.5e−4, Figure 3C). Neurons that failed migration were mostly located in the WM (Figures 3B–D). This result suggests that the β subunit-interaction contributes to the migration deficit caused by the I1166T mutation. The introduction of the double pore mutation L745P/E363A/E1115A, which virtually eliminates Ca^2+^-dependent gene expression (Servili et al., 2018), onto Cav1.2^I1166T^ elicited migration deficits with additional phenotypes (Supplementary Figure 2), suggesting that the normal level of Ca^2+^ influx is necessary for normal development.

### The Calmodulin Pathway but Not the Calcineurin Pathway May Be Responsible for the Migration Deficit by Cav1.2^I1166T^ Expression

**FIGURE 4:**
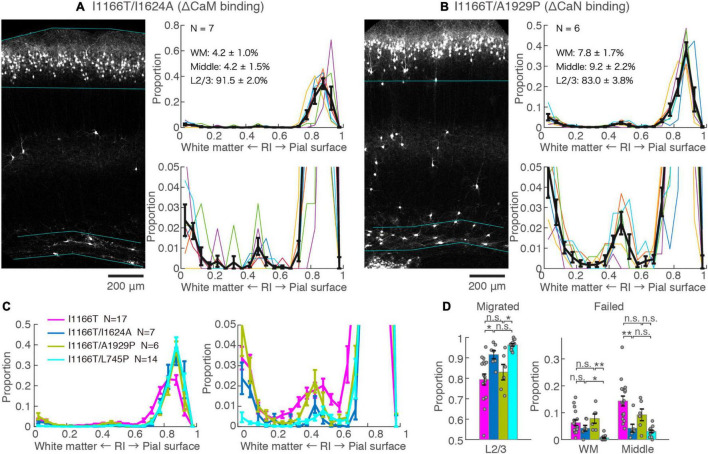
Blockade of CaM-binding but not CaN-binding rescues the migration deficit. **(A,B)** GFP images and histograms of the Cav1.2^I1166T/I1624A^ **(A)** and Cav1.2^I1166T/A1929P^ group **(B)**, as shown in Figure 2A. **(C)** Histogram of the mean ± SEM are merged for Cav1.2^WT^, Cav1.2^I1166T/I1624A^, Cav1.2^I1166T/A1929P^, and Cav1.2^I1166T/L745P^ groups. **(D)** Proportion of correctly migrated neurons (left) and that of migration-failed neurons located in WM and Middle (right). Independent values and mean ± SEM are shown. **P < 0.01; *P < 0.05; n.s., *P* ≥ 0.05.

Next, we examined the possible involvement of two major factors downstream of the Ca^2+^-influx, CaM and CaN, in Cav1.2I1166T-mediated migration defects. Both CaM and CaN bind to the C-terminal region of the Cav1.2 α_1_ subunit and modulate channel activity through the so-called Ca^2+^/CaM- and voltage-dependent inactivation (CDI and VDI, respectively) (Barrett and Tsien, 2008; Cohen-Kutner et al., 2012). CaM and CaN are also key players involved in Ca^2+^-dependent gene expression. First, we used the I1624A mutation, which is located at the C-terminal IQ motif of the Cav1.2 α_1_ subunit and impairs CaM binding (Zühlke et al., 1999). The vast majority of neurons expressing Cav1.2^I1166T/I1624A^ migrated normally (91.5 ± 2.0%, *N* = 7, Figures 4A,C,D). Although the fraction was slightly smaller than neurons expressing Cav1.2^I1166T/L745P^ (*P* = 0.12, Figure 4D), it was significantly larger than Cav1.2^I1166T^ expressing neurons (*P* = 0.026, Figure 4D). This result suggests that the migration deficit caused by the I1166T mutation was “rescued” by the blockade of the CaM interaction. In some samples, neuron numbers seemed to be lower than others (e.g., in Figure 4A). Therefore, we compared neuron densities between test groups (Supplementary Figure 3). Cav1.2^I1166T/I1624A^ group had slightly smaller density than I1166T group (*P* = 0.043). This result could be due to the disappearance of migration-failed neurons in Cav1.2^I1166T/I1624A^ group, or be due to the fact that the number of electroporated neurons by *in utero* electroporation generally varies widely between pups. Next, we used an A1929P mutation, which is located at the CaN-binding site of the C-terminal tail of the human Cav1.2 α_1_ subunit, which was shown to abrogate binding of CaN to the channel (Tandan et al., 2009; Xu et al., 2010). A considerable number of Cav1.2^I1166T/A1929P^-expressing neurons failed to migrate to the upper layer (17.0 ± 3.8%, *N* = 6, Figures 4B–D). The fraction of correctly migrated neurons was similar to that of Cav1.2^I1166T^-expressing neurons (*P* = 0.55, Figure 4D) and was significantly smaller than that of Cav1.2^I1166T/L745P^-expressing neurons (*P* = 0.012, Figure 4D). Thus, the migration deficit by I1166T was not “rescued” by the blockade of the CaN interaction.

### Cav1.2 I1166T Mutation Interrupts Callosal Axon Arborization in the Contralateral Cortex Through Calcium-Dependent Pathway and β Subunit-Interaction

**FIGURE 5:**
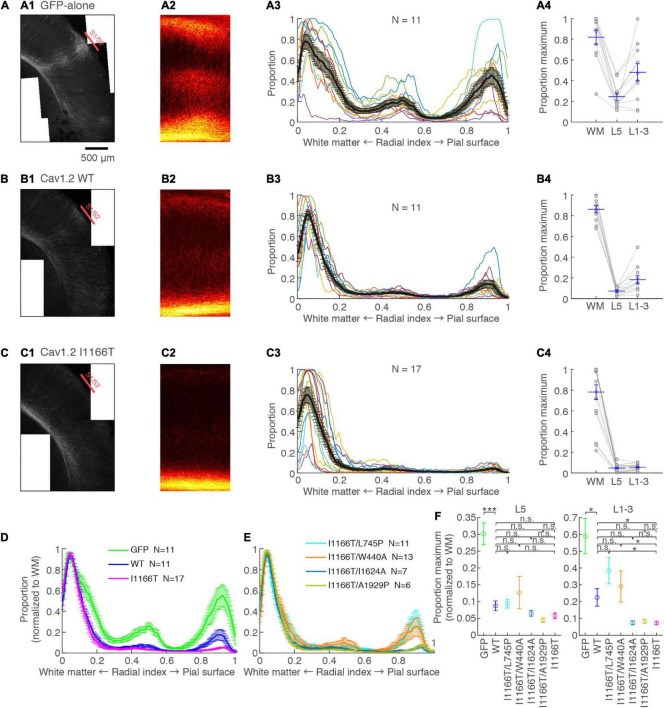
Axonal arborization in the contralateral cortex is affected by I1166T mutation, which is rescued by the blockade of calcium overload and β subunit-interaction. **(A)** Axonal arborization of the GFP-alone group. **(A1)** An example image of axonal arborization in the contralateral somatosensory area. **(A2)** Binarized images around the S1/S2 border of multiple samples were averaged and are shown. **(A3)** Proportion of pixels that have axonal signals are shown against the radial position. Colors for each sample: black, the mean ± SEM. **(A4)** Maximum proportion in the white matter (WM; Radial index = 0–0.1), layer 5 (L5; Radial index = 0.4–0.6), and upper layers (L1–3; Radial index = 0.8–1) of each sample; the mean ± SEM are shown. **(B,C)** Axonal arborization of Cav1.2^WT^- **(B)** and Cav1.2^I1166T^-expressing neurons **(C)** are shown as in **(A)**. **(D)** Proportions that were normalized with the maximum proportion in the WM are shown for the GFP-alone, Cav1.2^WT^, and Cav1.2^I1166T^ groups by the mean ± SEM. **(E)** Proportions for Cav1.2^I1166T/L745P^, Cav1.2^I1166T/W440A^, Cav1.2^I1166T/I1624A^, and Cav1.2^I1166T/A1929P^ groups are shown as in **(D)**. **(F)** Proportion in L5 (left) and L1–3 (right) for GFP, WT, and all mutation groups used in this study are shown by the mean ± SEM. ***P < 0.001; *P < 0.05; n.s., *P* ≥ 0.05.

L2/3 neurons provide most axons projecting to the contralateral hemisphere (Fame et al., 2011). Such callosal axons arbor densely at the S1/S2 border. The arborization was dense in the upper layers and L5 (Mizuno et al., 2007). These projection patterns were seen in samples with neurons expressing GFP-alone (Figure 5A). Axons clustered at the S1/S2 border and densely arborized in the upper layers and L5. Callosal axons of the Cav1.2^WT^ group were less arborized in the contralateral cortex and showed a moderate clustering in the S1/S2 border and in the upper layers and L5 (Figure 5B). On the other hand, callosal axons were largely diminished in the Cav1.2^I1166T^ group, and clustering was unclear (Figure 5C). The axonal GFP signal strength was significantly lower in the Cav1.2^WT^ group than in the GFP-alone group (L5, *P* = 8.2e−4; upper layer, *P* = 0.025), and in the Cav1.2^I1166T^ group than in the Cav1.2^WT^ group (L5, *P* = 0.70; upper layer, *P* = 0.023; Figures 5D,F). To further demonstrate that the I1166T mutation decreases callosal projection, we electroporated WT or I1166T α_1_ subunit-only (α_2_δ_1_ and β_3_ subunits were omitted). In this condition, exogenous α1 subunits should make a complex with endogenous α_2_δ_1_ and β_3_ subunits, thus the channel number increase would be alleviated. As we expected, both Cav1.2^WT^ α_1_-only and Cav1.2^I1166T^ α_1_-only groups showed increased callosal axon arborization compared with those expressing α_2_δ_1_ and β_3_ subunits together (*P* < 0.05 for all; Supplementary Figures 4A–E). The arborization of Cav1.2^I1166T^ α_1_-only group was highly less than that of Cav1.2^WT^ α_1_-only group (*P* = 0.0022 for both L5 and upper layer; Supplementary Figures 4D,E). Migration deficit was also worse in Cav1.2^I1166T^ α_1_-only group than Cav1.2^WT^ α_1_-only group (Supplementary Figures 4F–I). These results suggest that not only the Cav1.2 channel number but also the I1166T mutation decreases axon arborization in the contralateral cortex.

We analyzed contralateral projection for all mutant groups used for migration analyses. Whereas projection significantly decreased in all mutant groups compared with the GFP-alone group, the Cav1.2^I1166T/L745P^ and Cav1.2^I1166T/W440A^ group had a greater projection than the Cav1.2^I1166T^ group and was comparable to the Cav1.2^WT^ group (Figures 5E,F and Supplementary Figure 5). However, the Cav1.2^I1166T/I1624A^ group and the Cav1.2^I1166T/A1929P^ group failed to improve projection (Figures 5E,F and Supplementary Figure 5). These results suggest that Ca^2+^ influx and β subunit-interaction are responsible for the callosal projection deficit in Cav1.2^I1166T^-expressing neurons.

### Cav1.2 I1166T Mutation Does Not Affect Local Axon Arborizing in Layer 5 and Axonal Projection in the White Matter

**FIGURE 6:**
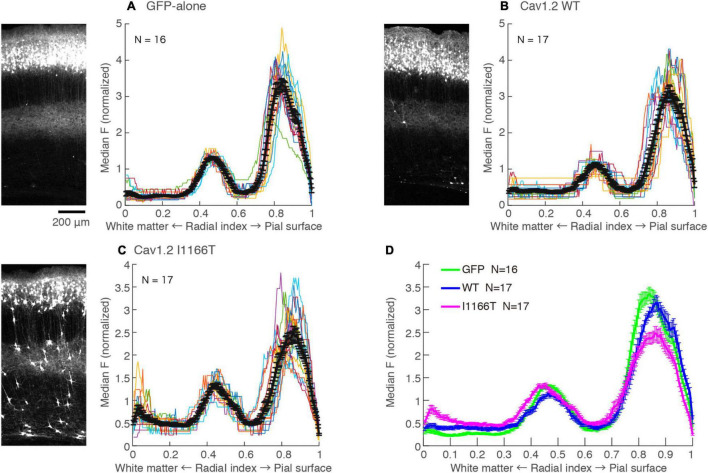
Local axonal arborization in the ipsilateral cortex is not affected by the expression of Cav1.2 mutants. **(A)** Left, An example GFP image in the electroporated hemisphere of GFP-alone group. A strong GFP signal was seen in the upper layer, representing cell bodies, dendrites, and axons of GFP-expressing neurons. A strong signal was also seen in the middle of the cortex, representing axons of neurons migrated to the L2/3. Right, the medians of GFP intensities are calculated along the tangential axis of the images. Colors, each sample. Black, the mean ± SEM. **(B,C)** GFP images and median intensities of Cav1.2^WT^ group **(B)** and Cav1.2^I1166T^ group **(C)**, as shown in **(A)**. Because migration-failed neurons were sparse in the middle of the cortex, their strong signals could be excluded and axonal signals could be obtained by calculating the medians. **(D)** The values of mean ± SEM of three groups are merged.

L2/3 neurons make axonal branches densely in layer 5 in the same ipsilateral cortical area (Mizuno et al., 2007). A strong axonal GFP signal was seen in the middle of the cortical depth in the GFP-alone group (Figure 6A; Radial index = 0.4–0.6), which may correspond to layer 5. Axonal GFP signal intensities in layer 5 were similar among the GFP-alone, Cav1.2^WT^, and Cav1.2^I1166T^ groups (Figure 6). Interestingly, migration-failed neurons in all Cav1.2 mutant-expressing groups tended to locate at the same depth (Supplementary Figures 6E,F). Layer 5 could attract not only L2/3 axons but also migrating L2/3 neurons in the developmental stage.

**FIGURE 7:**
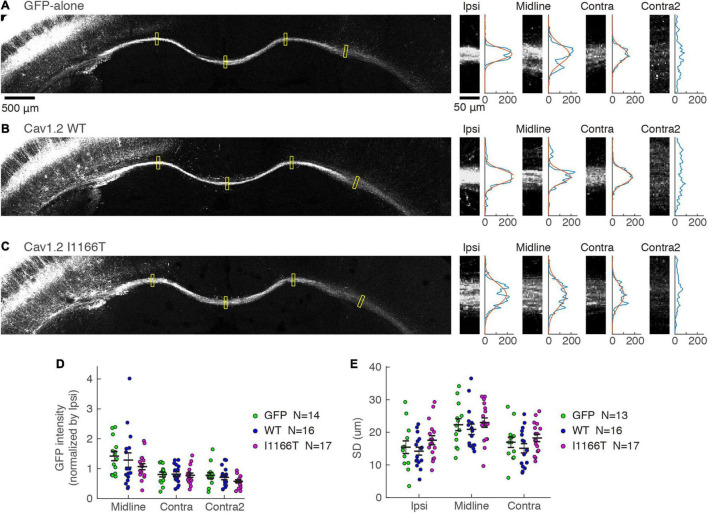
Axonal projection in the white matter is not affected by the Cav1.2 I1166T mutation. **(A)** Left, GFP images of white matter in the GFP-alone group. Yellow squares represent the four areas shown on the right. Right, GFP images of Ipsi, Midline, Contra, and Contra2 areas. Intensities of each area were averaged in the horizontal axis and shown on the right. Gaussian fit to the intensity distribution is shown in red. **(B,C)** Axonal projections of the Cav1.2^WT^ **(B)** and Cav1.2^I1166T^ **(C)** groups are shown as in **(A)**. **(D)** GFP intensities normalized by that of Ipsi are shown for each sample and the mean ± SEM for the GFP-alone, Cav1.2^WT^, and Cav1.2^I1166T^ groups. All the combination of groups had insignificant differences (*P* > 0.09). **(E)** Broadness of axon bundles in white matter, represented by the standard deviation of Gaussian curves, are shown for each sample and the mean ± SEM for the GFP-alone, Cav1.2^WT^, and Cav1.2^I1166T^ groups. All the combination of groups had insignificant differences (*P* > 0.1).

Next, we examined whether the mass of axons projecting in the white matter to the contralateral area differed. GFP intensities in the white matter were tested at the ipsilateral area, midline, and contralateral areas and were normalized with the intensity at the ipsilateral area. Similar signal strengths were seen in all locations among the GFP-alone, Cav1.2^WT^, and Cav1.2^I1166T^ groups (Figures 7A–D). Because the axonal position in the white matter is one of the factors regulating the callosal projection (Zhou et al., 2013), we also analyzed whether axon bundles broadened in the white matter in the Cav1.2^I1166T^ group. The broadness was also similar among the three groups (Figures 7A–C,E). Axonal projections in ipsilateral layer 5 and the white matter were similar for all mutant groups tested (Supplementary Figures 6A–E,G,H). These results suggest that signal cascades starting from Cav1.2 are less responsible for local axonal arborization and axonal elongation in the white matter but are crucial for axon invasion and arborization in the contralateral cortex.

## Discussion

In this study, we used mouse layer 2/3 cortical neurons as a model to demonstrate that expressing a disease-causing Cav1.2 mutant, I1166T, affected migration and axonal projections. Neuronal migration is crucial for the formation of the organized structure of the cerebral cortex, and migration deficits can be associated with ASD, seizures, and intellectual disability (Pan et al., 2019). Callosal axons connect the two cortical hemispheres; thus, they are important for higher cognitive functions. Alterations in the corpus callosum have been noted in patients with ASD and other psychiatric and developmental disorders (Egaas et al., 1995; Paul, 2011). Our findings suggest that brain development and neural circuit connectivity can be affected by the Cav1.2 I1166T mutation. Because patients with the I1166T mutation are rare and the possible abnormalities in the brain have been rarely studied, our study raises the possibility that patients with the I1166T mutation have defects in neuronal migration and/or callosal projections.

Regarding neuronal migration, the I1166T mutation altered migration, and the addition of the L745P mutation that decreases calcium influx improved migration. The addition of the I1624A mutation inhibiting CaM-binding to the α_1_ subunit partially recovered migration, whereas the A1929P mutation that inhibits CaN-binding to the α_1_ subunit did not improve migration. The most plausible explanation for these observations may be that the increase in calcium influx to neurons with the I1166T mutation altered Ca^2+^-dependent gene expression via Ca^2+^-CaM binding to the channel and impaired migration.

What is the mechanism underlying the migration recovery in Cav1.2^I1166T/I1624A^ in the current study? I1624 is responsible for binding to CaM; Ca^2+^-CaM binds to the C terminus of the Cav1.2 α_1_ subunit and modulates channel activity through the so-called Ca^2+^-dependent inactivation (CDI) and voltage-dependent inactivation (VDI) (Zühlke et al., 1999; Barrett and Tsien, 2008). Ca^2+^-CaM also regulates Ca^2+^-dependent gene expression (Deisseroth et al., 2003; Yap and Greenberg, 2018). The additional I1624A mutation may “cancel” the increased calcium influx caused by the I1166T mutation and/or normalize gene expression, by which the recovery of migration was observed in Cav1.2^I1166T/I1624A^. Consistent with this, it was shown that the I1624A mutation accelerated VDI in WT Cav1.2 (but not Cav1.2^G406R^; Barrett and Tsien, 2008). However, the inhibition of CaN-binding by the A1929P mutation has been shown to accelerate VDI of both the WT and G406R (Cohen-Kutner et al., 2012), but the Cav1.2^I1166T/A1929P^ mutation failed to improve migration. The mutation could also impair CaN-dependent gene expression. The effects of the A1929P mutation on channel modulation and possible gene expression regulated by CaN might not have been strong enough to rescue the phenotype caused by the I1166T mutation.

A previous study showed that expression of Cav1.2 G406R, which is the original mutation identified in patients with TS, caused migration defects in the mouse cerebral cortex (Kamijo et al., 2018). Callosal axon projections were not assessed in that study. Both the G406R and I1166T mutations are considered to be gain-of-function, though the channel property of Cav1.2 G406R and I1166T appears somewhat different (Boczek et al., 2015; Wemhöner et al., 2015). Whether the G406R mutant channel also impairs callosal axon projections is an intriguing question. Additionally, many other “gain-of-function” mutations associated with patients are reported in Cav1.2 (Ozawa et al., 2018; Zhang et al., 2018; Marcantoni et al., 2020). All these patients show LQTS type 8 in their hearts, but they exhibit various neurologic symptoms. It is important to test whether these gain-of-function mutant channels affect brain development and connectivity in the mouse model.

Critical roles of Ca^2+^ signaling in cortical neuron migration and axonal projections have been recognized (Ageta-Ishihara et al., 2009; Hutchins et al., 2011; Sutherland et al., 2014; Horigane et al., 2019). In this study, approximately 80% of neurons migrated to the correct layer (i.e., layer 2/3), although they were transfected with Cav1.2 I1166T. Nonetheless, the callosal projections were almost completely perturbed by the Cav1.2 I1166T expression. The expression of Cav1.2^WT^ only slightly affected migration but significantly affected callosal projections. These results suggest that axonal projection is more sensitive to Ca^2+^ overload than migration. The precise intracellular signaling mechanism by which the Cav1.2^I1166T^ channel leads to the deficits remains elusive, and further studies are required.

Interestingly, the overexpression of the Cav1.2 WT or all the mutants tested altered axonal growth in the contralateral cortex but not in the white matter (Figures 5, 7). Thus, axonal elongation and midline crossing were less perturbed, and defects were evident after callosal axons entered the gray matter. It has been shown that various players that regulate the intracellular calcium signaling (i.e., Cav1.2, Wnt, and TRP channels) are critically involved in callosal axon elongation, midline crossing, and axonal branching (Ageta-Ishihara et al., 2009; Hutchins et al., 2011). Among them, Cav1.2-mediated calcium signaling may play a part in the gray matter but not in the white matter.

Our results from experiments using Cav1.2^I1166T/W440A^ can be explained by two possible mechanisms. One is a crucial role of the interaction of Cav1.2 α_1_ subunit to the β subunit for membrane trafficking of the channels. In the literature, there are reports showing that W440A mutation affects channel trafficking and membrane expression of the WT channel (Obermair et al., 2010). If channel trafficking of the I1166T mutant is also dependent on the interaction to the β subunit through W440, then Cav1.2^I1166T/W440A^ would not be transported to the membrane and therefore show the “rescued” phenotypes. The other possible mechanism is the involvement of the β subunit-dependent signaling pathway (Servili et al., 2018). There are reports showing that W440A does not affect channel trafficking but influence downstream signaling (Berrou et al., 2002; Servili et al., 2018; Yang et al., 2019). Previous *in vitro* expression studies using HEK cells showed the relevance of the Ca^2+^-independent, β subunit-dependent pathway for the regulation of gene expression downstream of the Cav1.2 WT and G406R channels (Servili et al., 2018, 2020). This pathway recruits Ras/ERK/CREB to trigger c-Fos and MeCP2 activation. In the current study (Figures 3, 5), the W440A mutation might not have had major effect on channel trafficking in our experimental conditions. For example, if Cav1.2^I1166T/W440A^ was not transported to the membrane, we would have seen the migration and axonal projections of the Cav1.2^I1166T/W440A^ group to the level equivalent to the “GFP only” group. In fact, this was not the case; the result suggests that Cav1.2^I1166T/W440A^ would have been (at least partially) transported to the membrane and therefore have affected the migration and axonal projections. However, (1) to what extent the interaction with β subunit is essential for the membrane trafficking of the α_1_ subunit and (2) to what extent the specific amino acid residue W440 influences the channel trafficking and the downstream signaling remain to be elucidated in this study, and further studies are required.

There are some caveats in this study. First, this study used an overexpression strategy to reveal the effects of mutant channel expression on neuronal development. The strategy introduces mutant channels additional to the endogenously expressed channels; thus, changing the number of channels. Indeed, projections in the contralateral cortex were highly impaired in the WT group. Second, *in utero* electroporation, a gene transfection technique used in this study can introduce genes in a small number of cells in the brain. This is suitable for assessing the effect of expression at the cellular level, but linking the cellular phenotypes to behavior is difficult. A brain-wide knock-in strategy would be desirable for a more accurate study of the disease.

Ion channels play key roles in the formation and function of the nervous system (Medvedeva and Pierani, 2020; Smith and Walsh, 2020). Cav1.2 is critically involved in neural development. It also plays crucial roles in the heart, and patients with its mutations are relatively easily detectable by cardiac symptoms or testing. Indeed, many disease-causing, gain-of-function mutations are reported in Cav1.2, and their channel properties have been characterized in *in vitro* expression studies. Combining those studies with *in vivo* expression studies would facilitate our understanding of how each mutant channel alters the development, connectivity, and functions of the brain, which will eventually lead to a better understanding of the etiology of the neurological manifestations associated with the disease.

## Data Availability Statement

The original contributions presented in the study are included in the article/Supplementary Material, further inquiries can be directed to the corresponding author/s.

## Ethics Statement

The animal study was reviewed and approved by the Animal Experiments Committee and the Genetic Recombinant Experiment Safety Committee of Kagoshima University.

## Author Contributions

HN and YT designed the study. KS designed the plasmids expressing Cav1.2. NN-T and EK conducted the experiments and analyzed the data. NN-T and YT wrote the manuscript. All authors checked and approved the manuscript.

## Conflict of Interest

KS and HN were employees of Sumitomo Dainippon Pharma Co., Ltd. This study received funding from Sumitomo Dainippon Pharma Co., Ltd. The funder had the following involvement with the study: study design. The funder was not involved in data collection, analysis, interpretation, the writing of this article or the decision to submit it for publication. The remaining authors declare that the research was conducted in the absence of any commercial or financial relationships that could be construed as a potential conflict of interest.

## Publisher’s Note

All claims expressed in this article are solely those of the authors and do not necessarily represent those of their affiliated organizations, or those of the publisher, the editors and the reviewers. Any product that may be evaluated in this article, or claim that may be made by its manufacturer, is not guaranteed or endorsed by the publisher.
