# Supplementary material for: Involvement of Calcium-Dependent Pathway and β Subunit-Interaction in Neuronal Migration and Callosal Projection Deficits Caused by the Cav1.2 I1166T Mutation in Developing Mouse Neocortex
Source: Front Neurosci. 2021 Dec 8;15:747951. doi: 10.3389/fnins.2021.747951 (PMC8692569; doi:10.3389/fnins.2021.747951)
Supplement: Supplementary file 1 [file Data_Sheet_1.docx]

Supplementary Material

# Supplementary Data

Supplementary Material should be uploaded separately on submission. Please include any supplementary data, figures and/or tables. All supplementary files are deposited to FigShare for permanent storage and receive a DOI.

Supplementary material is not typeset so please ensure that all information is clearly presented, the appropriate caption is included in the file and not in the manuscript, and that the style conforms to the rest of the article. To avoid discrepancies between the published article and the supplementary material, please do not add the title, author list, affiliations or correspondence in the supplementary files.

# Supplementary Figures and Tables

For more information on Supplementary Material and for details on the different file types accepted, please see [here](http://home.frontiersin.org/about/author-guidelines#SupplementaryMaterial). Figures, tables, and images will be published under a Creative Commons CC-BY licence and permission must be obtained for use of copyrighted material from other sources (including re-published/adapted/modified/partial figures and images from the internet). It is the responsibility of the authors to acquire the licenses, to follow any citation instructions requested by third-party rights holders, and cover any supplementary charges.

## Supplementary Figures


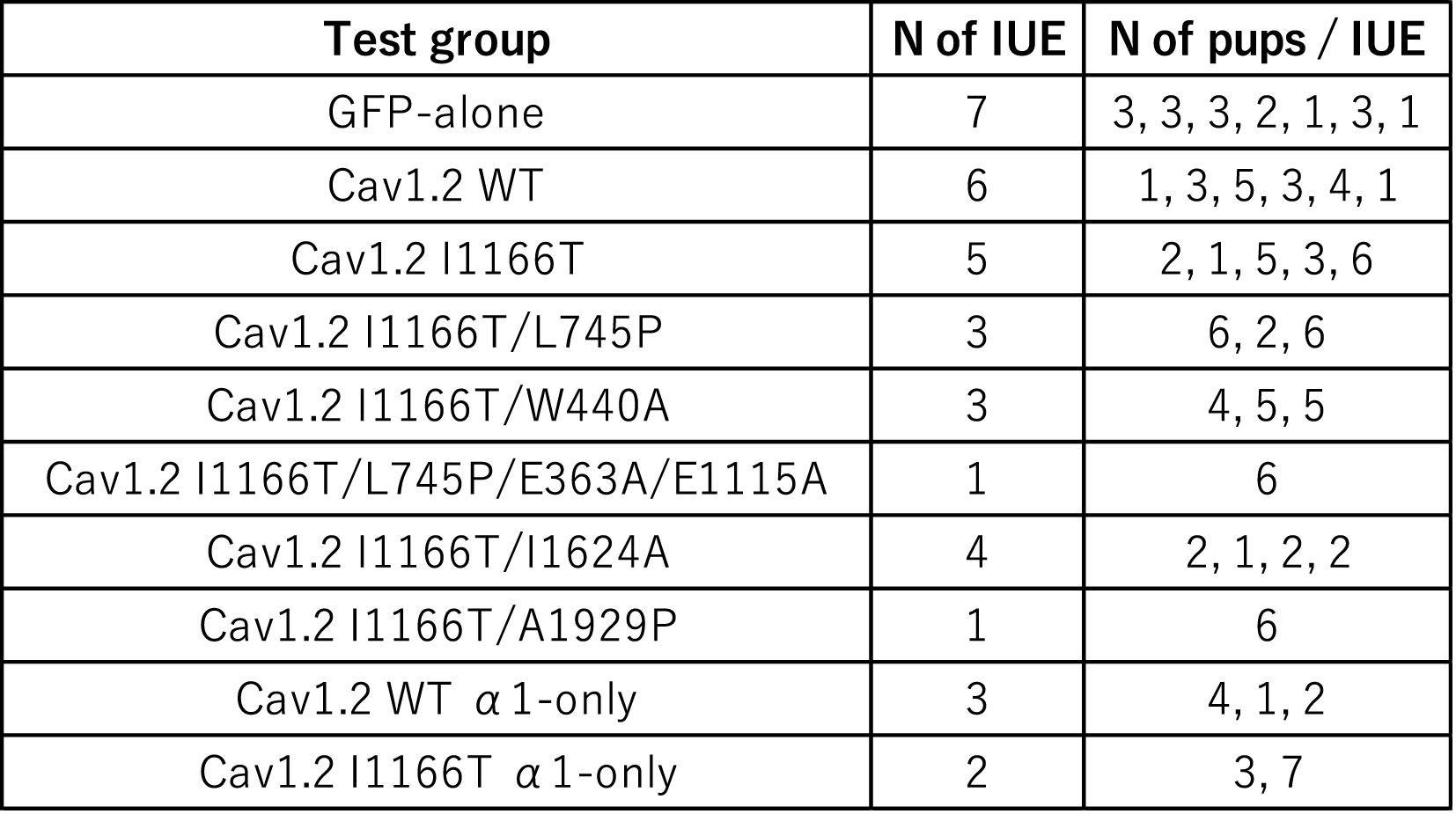


**Supplementary Figure 1.** The number of *in utero* electroporation (IUE) performed and the number of pups used from each IUE are shown for each test group.


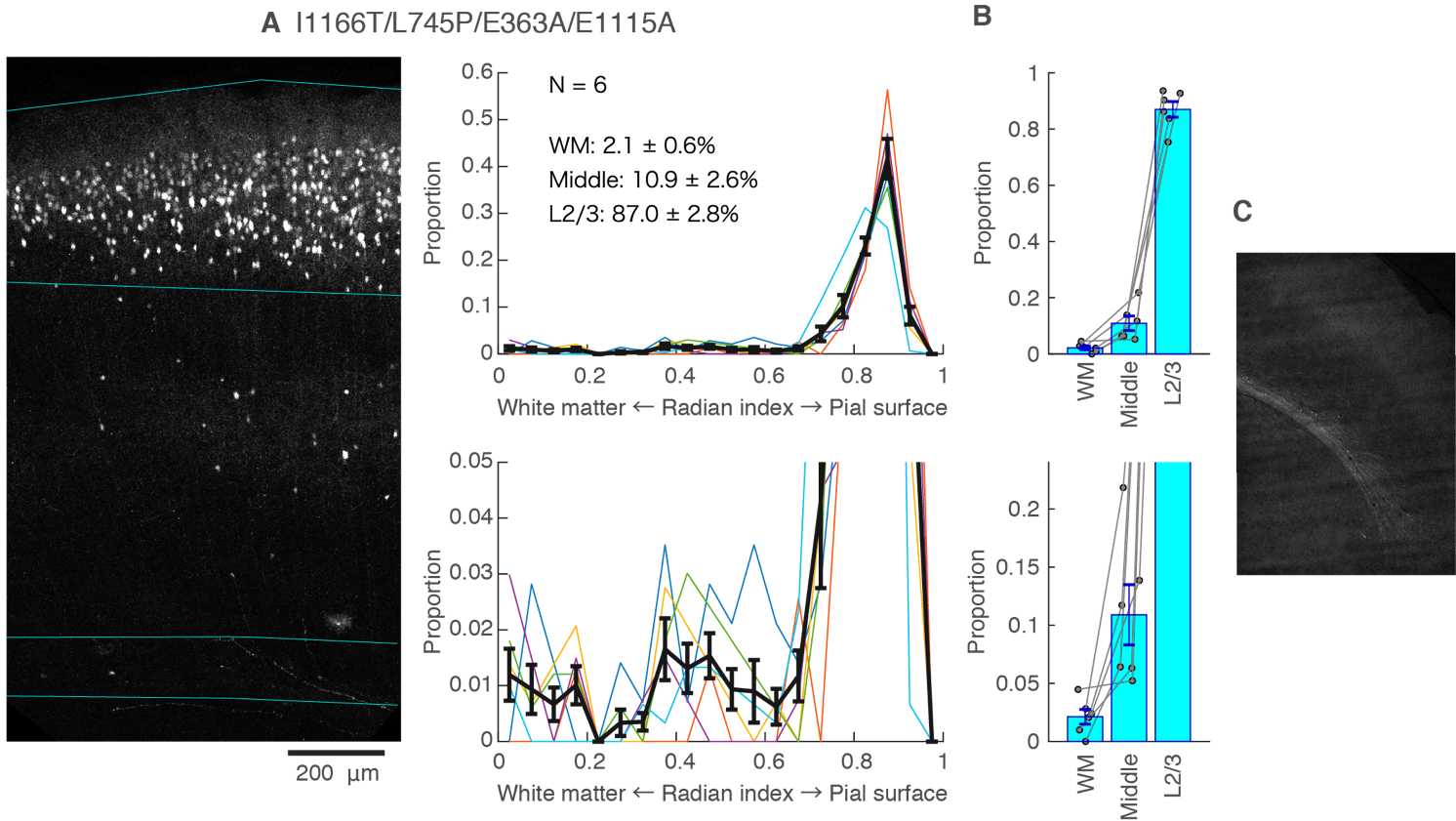


**Supplementary Figure 2.** The addition of double pore mutation L745P/E363A/E1115A onto Cav1.2^I1166T^ elicits migration deficits and axonal projections. **A.** An example GFP image and histograms of Cav1.2^I1166T/L745P/E363A/E1115A^ group, as shown in Figure 2A. The GFP signal intensity of the cell bodies, dendrites, and the axons in the layer 5 and the white matter were very weak. **B.** Proportion of correctly migrated neurons (L2/3) and that of migration-failed neurons (WM and Middle). Independent values and mean ± SEM are shown. Y-axis is enlarged and shown in the bottom. **C.** Though the axons were visible in the white matter in the contralateral area, axonal arborization in the contralateral cortex was absent.

**
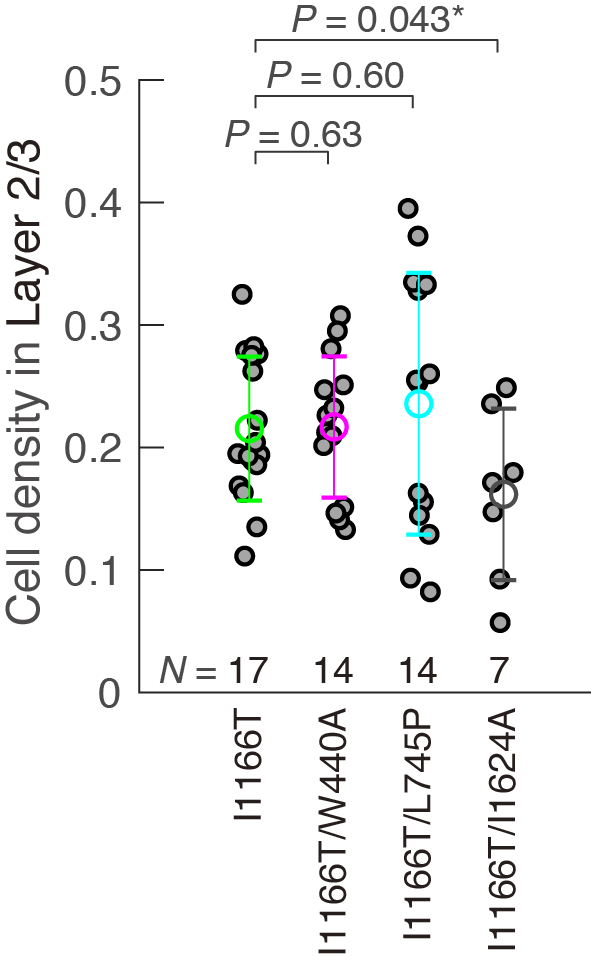
**

**Supplementary Figure 3.** The neuron densities were calculated by dividing the number of neurons in the analyzed area by the width (in micrometer) of the analyzed area. The density of both Cav1.2^I1166T/L745P^ and Cav1.2^I1166T/W440A^ groups were similar with that of Cav1.2^I1166T^ group, suggesting that the smaller number of migration-failed neurons seen by the addition of L745P or W440A mutations may be due to the migration recovery. On the other hand, Cav1.2^I1166T/I1624A^ group had slightly smaller density than I1166T group. This result could be due to the disappearance of migration-failed neurons in Cav1.2^I1166T/I1624A^ group or be due to the fact that the number of electroporated neurons by *in utero* electroporation generally varies widely between pups.


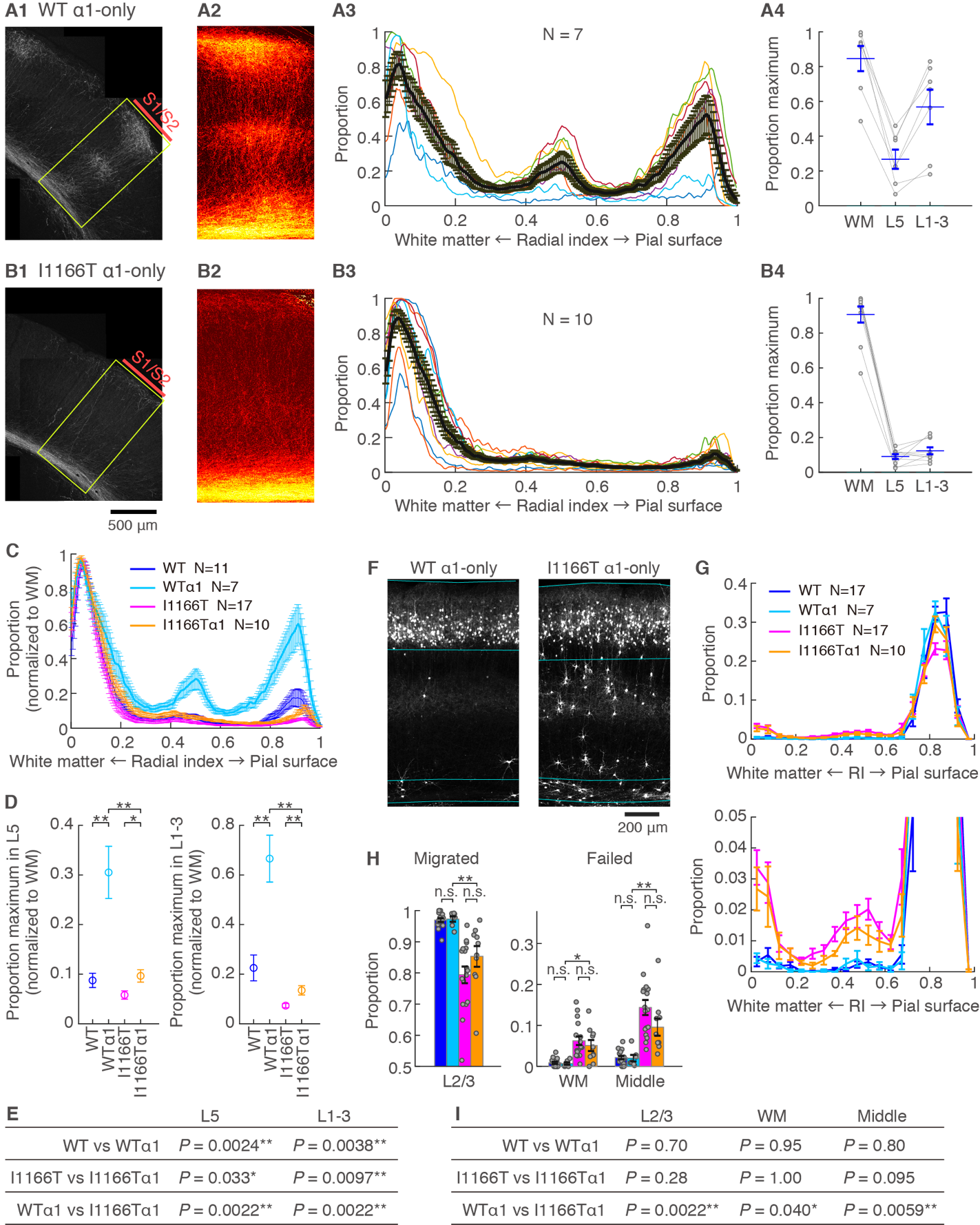


**Supplementary Figure 4.** The introduction of only α_1_ subunits alleviated the projection deficits and clearly revealed that I1166T mutation decreases callosal projection. **A**. Analysis of callosal projection for Cav1.2^WT^ α_1_-only group, as shown in Figure 5A. Axonal arborization in the contralateral somatosensory area (A1), binarized images around the S1/S2 border (A2), proportion of pixels that have axonal signals (A3), and maximum proportion in the (WM), layer 5 (L5), and upper layers (L1-3) (A4). **B.** Analysis for Cav1.2^I1166T^ α_1_-only group, as shown in A. **C.** Proportions that were normalized with the maximum proportion in the WM. **D.** Proportion in L5 (left) and L1–3 (right). **E.** *P* values in D are shown. **F**–**I**. Analysis of migration, as shown in Figure 2. **F.** GFP images of electroporated cortex**. G.** Histogram of the mean ± SEM among multiple samples. **H.** Proportion of correctly migrated neurons (top) and migration-failed neurons in WM and Middle (bottom). **I.** *P* values in H are shown.


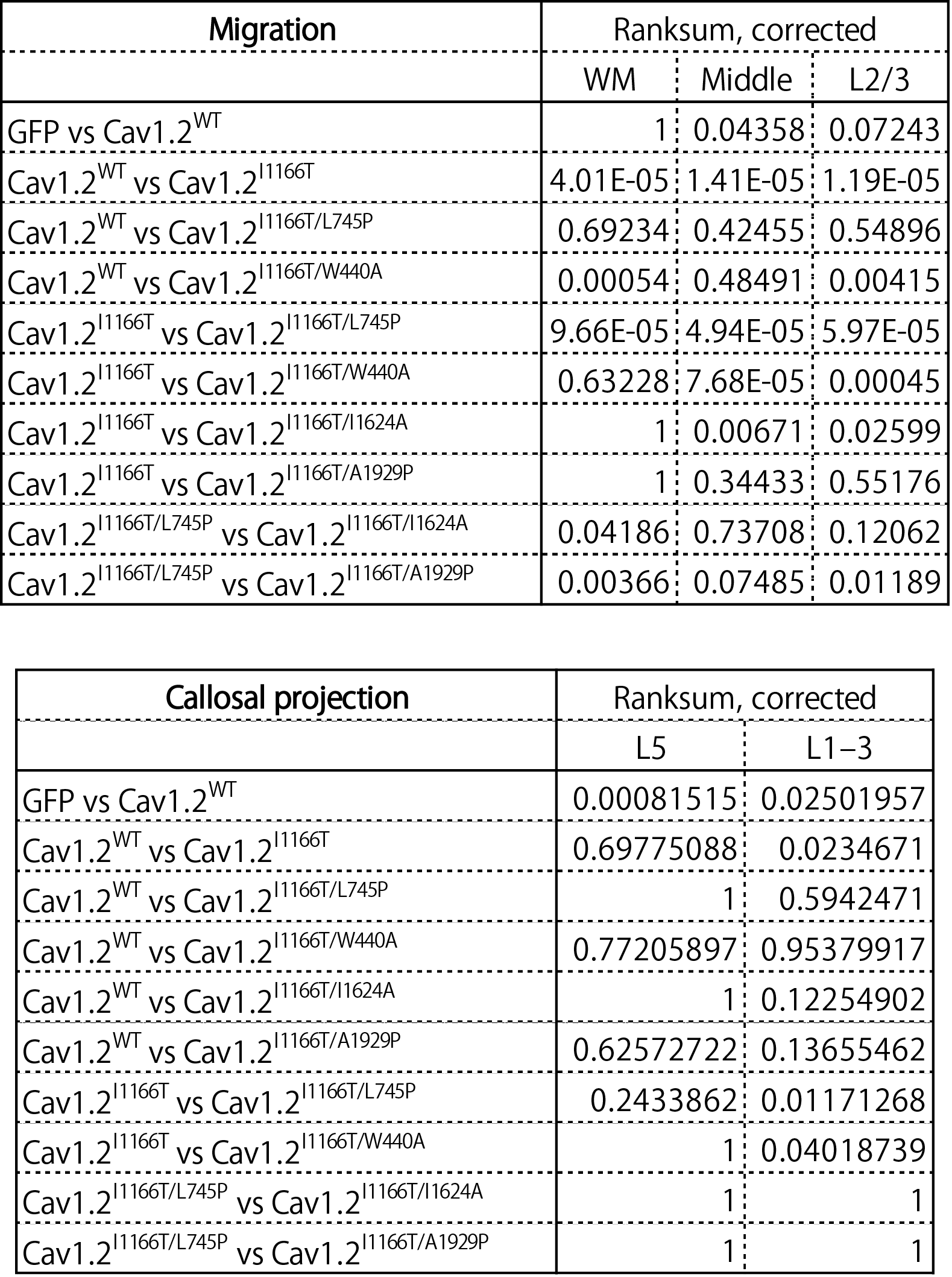


**Supplementary Figure 5.** *P* values of Mann-Whitney-Wilcoxon tests with Holm-Bonferroni correction are shown for the results of neuronal migration (top) and callosal projection (bottom). This table is related to Figure 2E, 3D, 4D, and 5F.


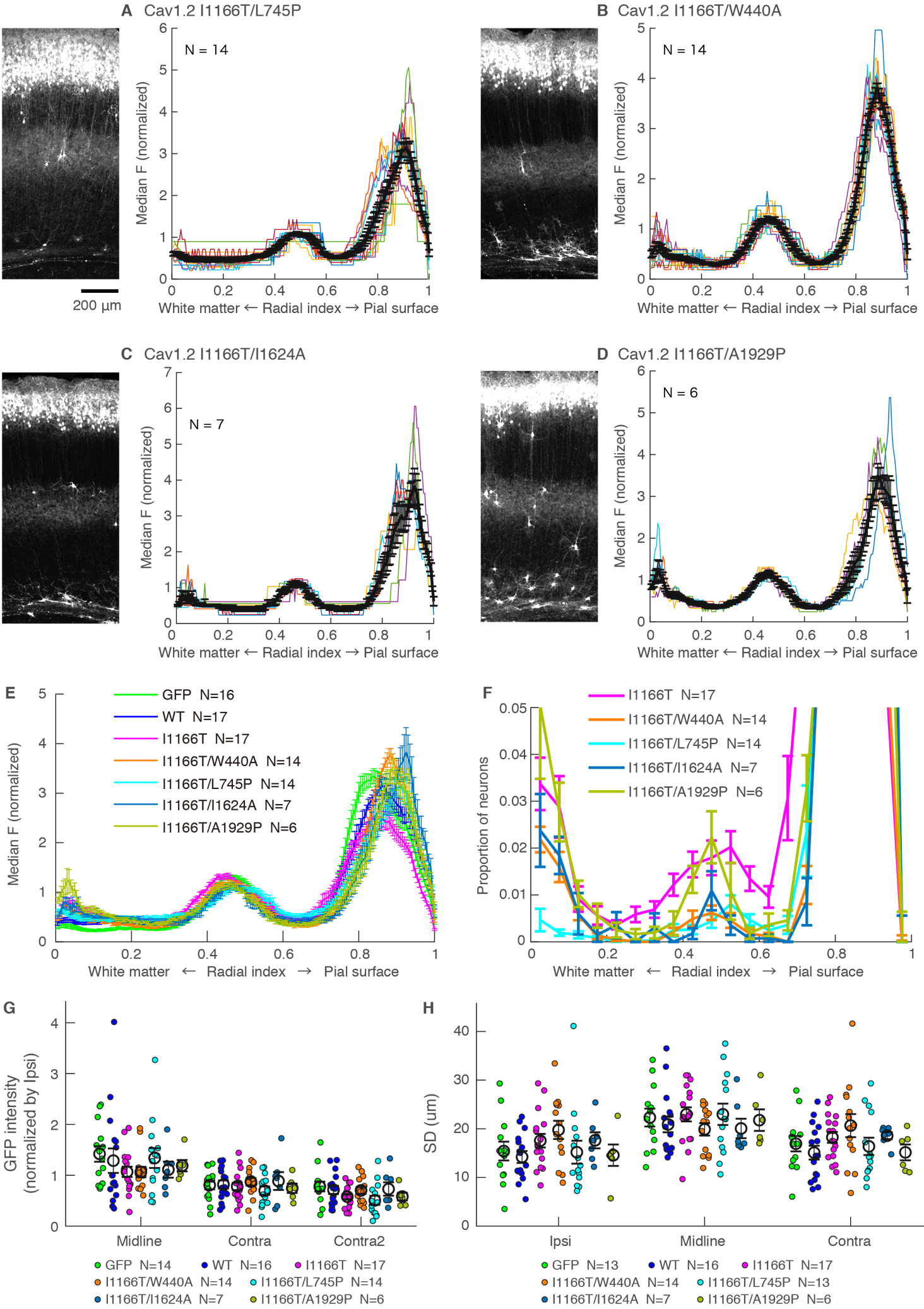


**Supplementary Figure 6.** Axonal projections in ipsilateral layer 5 and in the white matter were similar for all groups tested. **A–D.** An example GFP image in the electroporated hemisphere (Left) and median of GFP intensities calculated along the tangential axis of the images (Right) are shown for Cav1.2^I1166T/L745P^ (A), Cav1.2^I1166T/W440A^ (B), Cav1.2^I1166T/I1624A^ (C), and Cav1.2^I1166T/A1929P^ (D) groups. Colors, each sample. Black, mean ± SEM.**E.** Mean ± SEM of median of GFP intensities of GFP-alone, WT, and all mutants tested are merged. **F.** Proportion of neurons of mutant groups in which migration deficit was seen are merged. Migration-failed neurons tended to locate around layer 5 (radial index ≈ 0.5). **G, H.** GFP intensities (G) and broadness of axon bundles (H) in the white matter of GFP-alone, WT, and all mutants tested are shown as in Figure 7D and Figure 7E. All the combination of groups had insignificant differences (*P* > 0.1).
